# Another use of Foucher's flap

**DOI:** 10.4103/0970-0358.59299

**Published:** 2009

**Authors:** Ananta A. Kulkarni, Suhas V. Abhyankar, Rohit R. Singh, Ganesh S. Chaudhari

**Affiliations:** Department of Plastic Surgery, Dr D.Y.Patil Hospital & Research Centre, Nerul, Navi Mumbai, Maharashtra, India

Sir,

In respect to the article, Satish C, Nema S; First dorsal metacarpal artery islanded flap: a useful flap for reconstruction of thumb pulp defects. *Indian J Plast Surg* 2009;42:32-5, we would like to describe another use of Foucher's flap for palmar defect.

**Figure 1:**
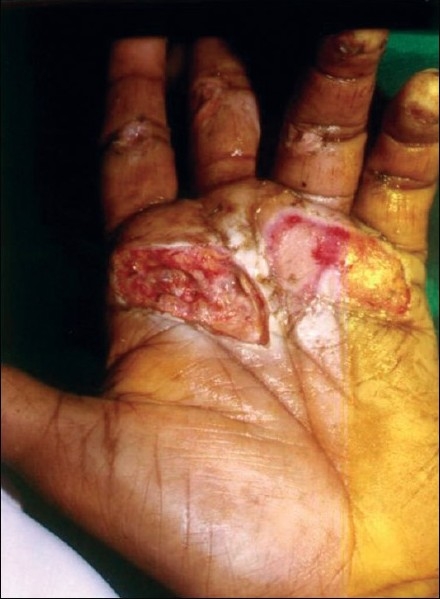
Palmar defect before surgery

**Figure 2:**
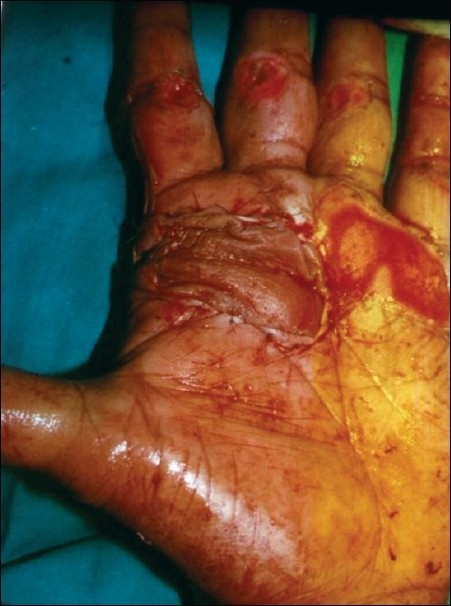
Post-surgery

**Figure 3:**
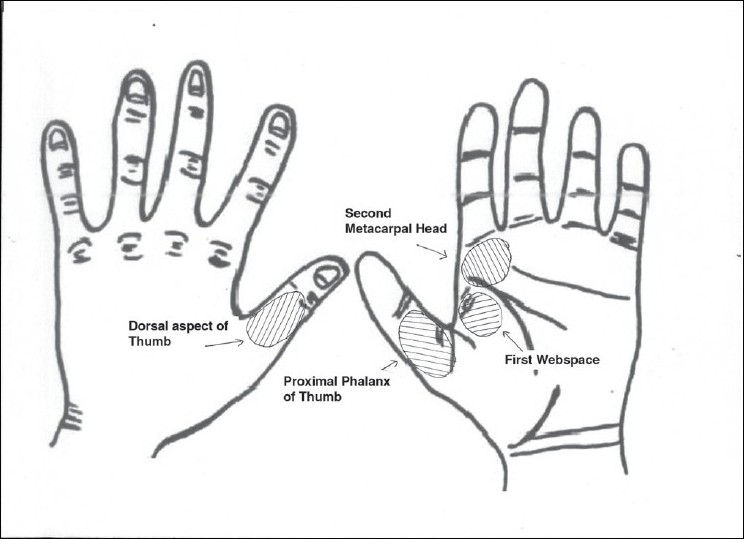
Arc of rotation of Foucher's flap

Traumatic palmar defects exposing tendons and nerves are usually covered by Groin flap and reversed radial forearm flap. We have done five Foucher's flaps up till now, four were for a thumb defect as described in this article and one was for a defect in the palm [Figures 1, 2], since Foucher's flap has a wide arch of rotation [Figure 3][1].

The venous drainage of the Foucher's flap is through the concomitant veins of the First dorsal metacarpal artery that are present in the areolar tissue in the pedicle of the island flap[2].

The reason for partial flap necrosis might be tension in the tunnel and postoperative oedema, if the primary closure of the donor defect is attempted. So the tunnel can be opened to avoid undue tension.

In our series of five patients, we have grafted the donor defect as well as the pedicle of the flap in two out of five patients, and in these two patients flaps survived completely.
